# Les Leçons du Programme de Lutte Contre les Vecteurs du Paludisme Par Aspersions Intradomiciliaires de DDT ou de Dieldrine dans la Zone Pilote de Bobo-Dioulasso: Échec ou Succès?

**DOI:** 10.48327/mtsibulletin.V9I9.66

**Published:** 2021-03-06

**Authors:** P. Carnevale, F. Fouque, F. Gay, S. Manguin

**Affiliations:** 1 Institut de recherche pour le développement (IRD), retraite administrative; 2 Special Programme for Research & Training in Tropical Diseases (TDR), World Health Organization, Avenue Appia 20, 1211 Geneva 27, Switzerland; 3 Hôpital Pitié-Salpétrière, 47 Bd de l'Hôpital, 75651, Paris, France; 4 HydroSciences Montpellier (HSM), Institut de Recherche pour le Développement (IRD), CNRS, Université Montpellier, France

**Keywords:** Paludisme, Lutte antivectorielle, Aspersions pariétales intradomiciliaires, DDT, Dieldrine, Évaluations entomologiques et parasitologiques, Histoire de la médecine, Zone pilote Bobo-Dioulasso, Burkina Faso, Afrique intertropicale, Malaria, Vector control, Indoor residual spraying, DDT, Dieldrin, Entomological and parasitological evaluations, History of medicine, Pilot Zone Bobo-Dioulasso, Burkina Faso, Sub-Saharan Africa

## Abstract

Pendant cinq ans, à partir de 1953, un grand programme d'aspersions pariétales intradomiciliaires s'est déroulé dans la zone pilote de Bobo-Dioulasso avec du DDT ou de la dieldrine (DLN) avec une évaluation conceptuellement entomologique et parasitologique [18].

Par rapport à la zone témoin, le DDT a induit une réduction d'environ 95% et 67% du taux de piqûres d'*Anopheles gambiae*, respectivement à l'intérieur et à l'extérieur des maisons. Mais du fait de son action irritante, le DDT a augmenté de cinq fois le coefficient d'exophagie de ce vecteur. La DLN n'a eu aucun impact sur le taux de piqûres d'*An. gambiae* aussi bien à l'intérieur qu'à l'extérieur, vu la résistance de l'espèce anophélienne à cet insecticide. L'indice sporozoïtique d'*An. gambiae* a été réduit de 96% dans les zones traitées au DDT et de 70% dans le secteur traité à la DLN.

Le DDT a réduit de 98% et de 91% le taux de piqûres d'Anopheles funestus, respectivement dans les maisons traitées et à l'extérieur. Avec la DLN, ces réductions ont été respectivement de 98% et 97%. L'indice sporozoïtique d'*An. funestus* a été réduit de 95% dans les zones traitées au DDT.

La lutte antivectorielle a permis de réduire la transmission due à *An. gambiae* et *An. funestus* de quelques 99,8% dans les villages traités au DDT par rapport aux villages témoins. La DLN a permis de réduire de 99,9% la transmission due à *An. funestus*, mais quasiment pas celle due à *An. gambiae* . La lutte antivectorielle basée sur les aspersions intradomiciliaires de DDT ou de DLN a permis de réduire de 99,9% la transmission des Plasmodium humains assurée par les deux vecteurs majeurs, *An. gambiae* et *An. funestus* dans les villages de la zone pilote.

Chez les enfants de 2–9 ans l'indice splénique a été de 84,3% (n= 979) en zone témoin et 44,4% (n = 8920) en zones traitées (différence: -47,3%), la prévalence plasmodiale a été de 60,6% (n = 946) en zone témoin et 38,0% (n = 7242) en zones traitées (différence: – 37%) mais les indices gamétocytiques sont restés aux mêmes niveaux (3,28%, n = 946 en zone témoin et 3,04%, n = 7242 en zones traitées) indiquant le maintien du « réservoir de virus » et des possibilités de transmission.

Par rapport à la zone témoin, « l'indice de contamination nouvelle » a été significativement moindre chez les nourrissons de 0-3 mois et de 4 à 6 mois dans les villages traités au DDT mais pas chez les nourrissons 7 à 12 mois démontrant que la lutte antivectorielle avait eu une certaine efficacité dans la prévention de l'infection plasmodiale mais « tous les nouveau-nés étaient infectés dans l'année », donc la transmission de *P. falciparum* n'avait pas été complètement stoppée.

Les aspersions intradomiciliaires avec le DDT ont été d'une grande efficacité dans la réduction de la transmission mais les vecteurs sont restés présents et la transmission, même fortement réduite, s'est maintenue de sorte que dans l'optique de l'éradication, le programme avait été considéré comme un « semi–échec » avec la décision d'adopter un changement complet de stratégie de lutte contre le paludisme et de se centrer sur la chimiothérapie de masse plutôt que sur la lutte antivectorielle.

Ces opérations de lutte antivectorielle d'aspersions pariétales de DDT peuvent être considérées comme un succès entomologique, avec ses limites indiquées par le maintien d'un certain niveau de transmission et des indices spléniques et plasmodiques ainsi que d'une incidence observée chez les nourrissons. Cinquante ans après, en l'absence de vaccins, cette recommandation de lutte intégrée et coordonnée contre les vecteurs et les Plasmodium est toujours d'actualité en bénéficiant, entre autres, des moustiquaires imprégnées et des traitements à base d'artémisine.

## Abstract (en)

During five years, from 1953, a village scale indoors residual spraying (IRS) was done in the pilot zone of Bobo-Dioulasso, Burkina Faso, with DDT or dieldrin (DLN) or even HCH with a conceptually both entomological and parasitological evaluation [18].

Compared to the control area, DDT induced an approximatively 95% and 67% reduction in the landing rate of *Anopheles gambiae*, respectively inside and outside human houses but due to its irritant action, DDT greatly increased their exophagic behaviour. However, DLN had no impact on the landing rate of *An. gambiae* either indoors or outdoors due to the already noticed resistance of this species to this insecticide. The sporozoitic index of *An. gambiae* was reduced by 96% in the DDT treated areas and by 70% in the DLN treated area.

DDT reduced the landing rates of Anopheles funestus by 98% and 91%, inside and outside treated houses respectively. With DLN, these reductions were 98% and 97%, respectively. The sporozoitic index of *An. funestus* was reduced by 95% in areas treated with DDT.

Thus, vector control has reduced malaria transmission due to the two main vectors, *An. gambiae* and *An. funestus*, by some 99.8% in DDT treated villages compared to control villages. DLN reduced transmission from *An. funestus* by 99.9%, but almost not from *An. gambiae* . Overall, the implementation of vector control based on indoor residual spraying with DDT or DLN reduced by 99.9% the transmission of human Plasmodium in the villages of the pilot zone and therefore the program can be considered as entomologically successful.

In children aged 2–9 years (target group for endemicity indices) the splenic index was 84.3% (n = 979) in the control area and 44.4% (n = 8920) in the treated areas (difference -47.3%), the plasmodial prevalence was 60.6% (n = 946) in the control zone and 38.0% (n = 7242) in the treated zones (difference - 37%) but the relatively high level of plasmodic or splenic index in treated villages showed that transmission was maintained at such a level that the program could be considered as a “semi-failure”.

Besides, the gametocytic indices remained at the same levels (3.28%, n = 946 in the control zone and 3.04%, n = 7242 in the treated zones) indicating the maintenance of the “reservoir of parasites” and the remaining possibilities of transmission.

Compared to the control area, the index of new contamination was significantly lower in infants 0-3 months and 4 to 6 months in DDT treated villages but not in infants 7 to 12 months demonstrating that the control vector had some efficacy in the prevention of plasmodial infection but “all newborns were infected within one year” demonstrating that *P. falciparum* transmission was not completely stopped.

In spite of its striking drop, the transmission was not fully stopped, and the programme was considered as a “semi-failure” or even a “failure” and inducing a complete shift in malaria control policy from vector control to mass drug chemotherapy (with several drugs, chloroquine, primaquine, pyriméthamine etc) without complete stop of transmission either. In fact, such vector control operations by DDT may have different analysis; in one side they can be considered an entomological success but, in another side, the actual reduction of plasmodic and splenic indices was not enough to be considered as successful. It was clear that both vector and parasite must be implemented in an integrated programme taking care of insecticide and drug resistance. Nevertheless, such programme, even not as successful as expected, could be considered as encouraging and not “disappointing” as it was. Important lessons can be learned from such large-scale field trial in spite of several methodological and operational issues.

## Introduction

La première conférence sur le paludisme en Afrique équatoriale s'est tenue à Kampala (Ouganda) du 27 novembre au 9 décembre 1950 pour discuter de l'éradication du paludisme par le traitement des maisons avec le DDT, dont les propriétés insecticides avaient été découvertes par Muller en 1939 [28] pour la société Geigy à la recherche de produits contre les mites (insectes microlépidoptères) des vêtements.

Deux « écoles » se sont alors formées: les « interventionnistes », avec une volonté d'action immédiate et les « conservateurs », demandant d'avoir davantage d'informations scientifiques sur l'immunité avant de lancer des grands programmes de lutte antivectorielle qui pourraient altérer l'immunité des populations en zones hyperendémiques. Une des recommandations de cette conférence a été que les gouvernements responsables de l'administration des territoires en Afrique entreprennent la lutte contre le paludisme par les méthodes modernes aussi vite que possible, quel que soit le niveau d'endémicité et sans attendre les résultats d'autres expérimentations [45].

Le Comité d'experts OMS réunis à Kampala juste après la conférence accepta ces recommandations [8] considérant qu'il n'était pas déraisonnable de commencer la planification de l'éradication du paludisme dans le monde [32] avec un programme de lutte antivectorielle, en trois étapes: attaque (3-4 ans), consolidation (3 ans et plus), maintenance, permettant d'obtenir l'arrêt complet de la transmission et l'éradication de la maladie palustre en un laps de temps relativement court [31].

Pour obtenir des informations scientifiques pertinentes et fiables sur les problèmes éventuels de la lutte antivectorielle en zones d'endémie d'Afrique sub-saharienne, il avait été décidé de mettre en oeuvre différents programmes pilotes dans des zones écologiquement différentes comme, notamment, les projets de Pare-Taveta en Tanzanie et au Kenya [9, 10, 40, 41, 42, 43, 44], de Thiès au Sénégal, de Yaoundé et Douala au Cameroun [2, 22] et de Bobo-Dioulasso en Haute-Volta, aujourd'hui Burkina Faso [18].

Ces programmes avaient pour objectif de tester la possibilité d'obtenir l'arrêt définitif de la transmission avec des opérations d'aspersions pariétales intradomiciliaires (essentiellement de DDT) et, ainsi, l'éradication de la maladie en un laps de temps défini. L'emploi des insecticides à action rémanente dans les campagnes antipaludiques ne visait pas la destruction de tous les anophèles, mais tendait seulement à réduire leur taux quotidien moyen de survie pour les empêcher d'atteindre un âge épidémiologiquement dangereux [23]. On cherchait à interrompre la chaîne épidémiologique de la transmission du paludisme au niveau du vecteur, avant qu'il ne se pose à l'intérieur des maisons pour son repas sanguin [17]. Les projets n'ayant pas obtenu l'arrêt complet de la transmission par cette méthode ont alors été considérés comme des échecs, même si une forte réduction de la transmission, et de la maladie, avait été obtenue.

Cette conclusion de « l'inefficacité du DDT » a conduit à un changement de stratégie, de la lutte antivectorielle à la chimiothérapie de masse.

Plus de 50 ans plus tard, dans le cadre d'un programme de réduction progressive de la transmission de la maladie pour obtenir son élimination [30], ces opérations de lutte antivectorielle auraient été considérées comme une certaine, et encourageante, réussite ainsi que le démontre une nouvelle analyse des résultats entomologiques et parasitologiques de la zone pilote de Bobo-Dioulasso. Sa conclusion négative s'est traduite par un arrêt de telles opérations de lutte antivectorielle et la recherche de nouveaux insecticides en remplacement du DDT et autres organochlorés notamment avec la construction de la station expérimentale et les cases-pièges de Soumousso (J. Hamon, com. pers.).

Il est historiquement intéressant de noter que c'est justement dans ces cases pièges de cette station que, 30 ans plus tard, ont été expérimentées les premières moustiquaires imprégnées de perméthrine [6] avec les premières évaluations épidémiologiques dans les environs de Bobo-Dioulasso [3, 34] procurant des résultats intéressants, notamment la réduction de 50% de la morbidité palustre. Cette nouvelle méthode de lutte a ensuite été utilisée avec succès dans de nombreux pays, avec des conditions écologiques et épidémiologiques différentes. De vastes programmes mis en oeuvre notamment en Gambie, au Ghana et au Kenya à l'initiative de TDR, ont montré une réduction de la transmission palustre (80%), de la morbidité (50%) et de la mortalité générale infanto-juvénile (17%) [21].

Les opérations menées dans la zone pilote de Bobo-Dioulasso ont aussi clairement révélé le problème des résistances des vecteurs aux insecticides avec celle, déjà connue à l'époque, d'*An. gambiae* à la dieldrine (DLN). Ce problème reste d'actualité avec les résistances actuelles aux pyréthrinoïdes utilisés pour l'imprégnation des moustiquaires.

## La Zone Pilote de Bobo-Dioulasso

### Présentation de la zone

La zone pilote de Bobo-Dioulasso (11°11'N; 4°20'O) (annexe 1) a été créée en 1952 après un accord entre le gouvernement français, l'OMS et l'UNICEF.

La situation écologique est détaillée dans le numéro spécial des Cahiers ORSTOM consacré à ce projet [18]. Le climat est de type soudanien, avec une longue saison sèche (novembre à avril) entrecoupée d'une petite saison des pluies en février (dite pluie des mangues), entraînant une petite poussée saisonnière de transmission (et l'association populaire des mangues et du paludisme). La végétation est celle d'une savane boisée, mais aux alentours des gros villages, tous les arbres ont été coupés pour servir de bois de feu et de charpente tandis que les feux de brousse font disparaître la végétation herbacée.

Les données météorologiques de l'époque indiquent la présence de pluies de mars à décembre 1957 avec la poussée saisonnière habituelle observée de juin à octobre

L'humidité relative variait entre < 20% en saison sèche à > 80-90% pendant la grande saison des pluies. La température moyenne était d'environ 28 °C avec un minimum en janvier (environ 18 °C) et un maximum en mars (environ 36 °C) et de grandes variations d'une année à l'autre, notamment la durée de la saison dite froide (décembre-février).

### Insecticides utilisés et opérations de pulvérisations pariétales intradomiliciaires

Les détails des produits et des opérations mises en oeuvre sont indiqués en annexe. Les opérations ont débuté le 1^er^ avril 1953.

## Méthodes D'enquêtes

### Enquêtes entomologiques et indicateurs

À l'époque, le choix des villages a été fait en fonction de leur « représentativité de la diversité de la zone pilote » [18] et non par randomisation.

Plusieurs méthodes de capture ont été utilisées: récoltes manuelles le matin de la faune résiduelle dans les maisons, suivies d'aspersions de pyréthrine; captures de nuit sur sujets humains, à l'intérieur et à l'extérieur des maisons; moustiquaires pièges; cases pièges; pièges de sortie; faune extérieure avec des abris artificiels.

Les trois premières années, de janvier 1953 à novembre 1956, « la seule méthode de capture couramment employée fut la recherche des moustiques adultes au repos le matin dans les habitations » [18], mais on connait les limites de cette technique dans le cas de moustiques à tendance exophile exophage et donc pour évaluer l'impact d'un programme de lutte visant à réduire la densité agressive pour l'Homme. À partir de septembre 1956, les captures de nuit ont commencé sur sujets humains « selon le système adopté par Haddow [12] à Bwamba et amélioré par Roberts et O'Sullivan [38] en Australie ». Les captureurs étaient placés à l'intérieur et à l'extérieur des habitations des villages témoins ou traités et prenaient les moustiques directement sur leurs jambes partiellement dénudées, car « les anophèles piquent presque exclusivement au-dessous du genou ».

Il n'y a pratiquement pas eu d'enquêtes entomologiques préliminaires dans les villages concernés et, pour diverses raisons, ces captures n'ont pas toutes été faites aux mêmes périodes dans les trois zones considérées (selon les traitements insecticides ou non). Il est intéressant de remarquer que la construction de cases-pièges dans les villages témoins a permis d'étudier les comportements naturels des vecteurs, notamment leur exophilie, en dehors de toute intervention de lutte.

Pour cette nouvelle analyse, nous n'avons considéré que les résultats des classiques captures de nuit sur sujets humains faites à l'intérieur et à l'extérieur des maisons des zones traitées ou non, simultanément aux mêmes périodes pour permettre une comparaison de type « ici et là ».

En outre, des tests de sensibilité aux insecticides avaient mis en évidence « la présence du gène de la résistance au Dieldrin » chez *An. gambiae* Giles dans les zones non traitées à la DLN de la région de Bobo-Dioulasso [15, 16].

Les anophèles capturés ont été morphologiquement déterminés puis disséqués pour établir leur âge physiologique; à l'époque, la méthode devenue classique de l'examen des trachéoles ovariens [7] n'existait pas et la détermination de l'âge physiologique a été basée sur l'examen des spermathèques et des follicules ovariens (« non fécondée et follicules au stade I de Christophers = femelle très jeune, moins de 48h »; versus « fécondée et follicules à un stade de maturation plus avancé = femelle pas très jeune »), puis de dissection complète du tractus ovarien [33] pour apprécier les femelles nullipares et pares. Mais la méthode a été abandonnée (difficultés de réalisation et perte de temps). La détermination de l'âge moyen des populations anophéliennes a été faite à partir des indices sporozoïtiques et d'une série d'abaques.

L'infectivité des vecteurs a été étudiée par l'examen microscopique des glandes salivaires à la recherche des sporozoites.

Outre les indicateurs classiques (taux de piqûres, indice sporozoïtique), il a été calculé un intéressant « coefficient d'exophagie » = « nombre de spécimens capturés sur sujets humains à l'extérieur/nombre de spécimens capturés sur sujets humains à l'intérieur des habitations ».

### Indicateurs parasitologiques et cliniques

Un recensement des enfants de moins de 10 ans a été fait dans chaque village témoin et « la plupart » des villages traités avec les classes d'âge suivantes:

- nourrissons de 0-12 mois divisés en trois sous-classes: 0-3 mois inclus; 4-6 mois inclus et 7-12 mois inclus;
- enfants de 13 mois-9 ans, divisés en trois sous-classes: 13-24 mois inclus; 2-4 ans inclus et 5- 9 ans inclus;
- la classe adulte correspond à toutes les personnes de plus de 10 ans.

En fonction de la taille du village, soit tous les enfants ont été examinés, soit un échantillon de taille variable d'enfants de 2–9 ans a été examiné avec deux indicateurs: la splénomégalie (« évaluée par la cotation de Hackett… sur les enfants debout ») et la prévalence plasmodiale (éléments asexués et gamétocytes) établie par la classique confection et examens au microscope optique des gouttes épaisses. La détermination des espèces plasmodiales a été faite par lecture de frottis.

Outre les habituels indices plasmodiques et gamétocytiques, en regroupant toutes les espèces plasmodiales (« *P. falciparum*, très abondant, *P. malariae* peu abondant, *P. ovale* très rare »), il a été calculé, pour chaque mois, un « indice de contamination nouvelle » ou ICN qui témoigne, à l'intérieur d'un groupe d'âge donné, des infections nouvelles qui ont été décelées au cours du mois. Il est calculé selon la formule: ICN = (nombre des nouveaux positifs x 100)/nombre de cas antérieurement indemnes. Cet ICN peut, en fait, être considéré comme une mesure de l'incidence mensuelle.

Il a aussi été établi un « indice cumulatif » (I.cum) « qui paraît mieux rendre compte du degré d'infection d'un groupe d'âge déterminé que l'indice parasitaire simple du mois considéré ». Cet indice cumulatif est obtenu avec la formule: I.cum = (total de tous ceux qui sont, ou ont été, positifs x 100)/nombre de nourrissons recensés.

Deux remarques peuvent être faites sur ces indices de contamination. D'abord les responsables du projet les considèrent « d'usage courant en trypanosomiase ». Ensuite, ce principe d'analyser l'évolution de la parasitémie de « négative » à « positive » dans un laps de temps considéré a été utilisé dans le modèle mathématique de Muench [27] repris dans le projet Garki [25] pour estimer le taux d'incidence (« incidence rate »). Le même principe a été utilisé pour la démarche inverse, c'est-à-dire de positif à négatif pour calculer le taux de guérison (ou « recovery rate »), dans les enquêtes longitudinales pour évaluer l'efficacité des opérations de lutte antivectorielle par aspersions intradomiciliaires de propoxur.

### Tests statistiques

Les données originelles avaient été analysées avec le test classique du χ^2^. Certaines ont été reprises dans le présent document avec le logiciel Graph Pad, puis analysées avec le test non paramétrique de Mann-Whitney qui permet de comparer les distributions et les médianes des effectifs tels qu'exposés dans le document publié. Le seuil de significativité était de p = 0,05.

## Résultats

### Résultats entomologiques

Vingt-trois espèces d'anophèles ont été capturées et 23621 glandes salivaires ont été disséquées; leur examen a montré que les principaux vecteurs étaient *An. gambiae* s.l. (avec un indice sporozoïtique d'environ 2%) et *An. funestus* (avec un indice sporozoïtique d'environ 1%). Mais des sporozoites ont aussi été observés chez *An. coustani, An. nili, An. flavicosta* et *An. brohieri* (tableau 1).

**Tableau I:** Dissections et indices sporozoïtiques observés dans la zone pilote de Bobo-Dioulasso Dissections and sporozoitic index noticed in the pilot zone of Bobo-Dioulasso

Espèces			
	GS +	GS disséquées	%
*An. coustani*	2	1712	0,12%
*An. nili*	5	2980	0,17%
*An. funestus*	121	11126	1,09%
*An. flavicosta*	2	1409	0,14%
*An. brohieri*	1	195	0,51%
*An. gambiae*	115	6199	1,86%
Total	246	23621	1,04%

GS+ = nombre de glandes salivaires avec des sporozoites

GS = nombre de glandes salivaires disséquées

% = pourcentage de glandes salivaires positives

Il a été noté des sporozoites chez *An. coustani* qui « semble très agressif vis-à-vis des humains, mais pique surtout à l'extérieur des habitations » et « participe probablement au maintien de la transmission du paludisme en zone traitée, son exophagie prononcée et son exophilie totale le mettant entièrement à l'abri des aspersions domiciliaires »; *An. nili* est surtout présent dans les localités près des grandes rivières à débit permanent, « il est très agressif vis-à-vis de l'Homme et semble piquer aussi volontiers à l'intérieur qu'à l'extérieur… il manifeste une exophilie à peu près totale… *An. nili* est l'un des responsables majeurs du maintien de la transmission dans les régions traitées ». Concernant *An. flavicosta*, « il est assez agressif vis-à-vis de l'Homme au moment où il est très abondant et attaque alors assez volontiers à l'intérieur des habitations non traitées. *An. flavicosta* doit jouer un rôle négligeable vis-à-vis de ceux d'*An. gambiae* et d'*An. funestus* dans les villages non traités » [18]. L'infection salivaire d'*An. brohieri* est sujette à caution (problème de détermination).

Dans ces conditions, les présentes analyses complémentaires des données entomologiques ont concerné les deux principales espèces vectrices: *An. gambiae* s.l. et *An. funestus*. Il est noté pour *An. gambiae* que « sa fréquence maximum se situe généralement pendant la saison des pluies bien que dans certains cas particuliers, il puisse être plus abondant en fin de saison sèche que pendant la saison des pluies »; et pour *An. funestus*, « il est présent en toute saison, sa fréquence maximum se situe durant les deux derniers mois de la saison des pluies et il est particulièrement rare au début des grandes pluies » [18].

### Évolution d'*An. gambiae*

#### Densités agressives (ma)

Les nombres moyens de piqûres d'*An. gambiae* par Homme et par nuit dans les secteurs témoins et traités de la zone pilote ont été recalculés en tenant compte des différentes périodes de captures simultanées et regroupés dans le tableau 2.

**Tableau II:** Nombres moyens de piqûres (ma) d'*An. gambiae/*Homme/nuit dans les villages des 3 secteurs, témoins, traités au DDT ou à la DLN Average number of landing rates (ma) of An. gambiae/human being/night in the villages of the 3 sectors: control; DDT treated or DLN treated

Secteurs		Témoin		DDT		DLN	
Périodes		Int.	Ext.	Int.	Ext.	Int.	Ext.
nov.56- fév.58*	ma	1,261	1,273				
	écart type	1,679	1,594				
mai 57- fév.58**	ma	1,907	1,861	0,106	0,654		
	écart-type	1,853	1,775	0,120	0,546		
mai 57- déc.57***	ma	2,372	2,322	0,133	0,757	6,435	6,125
	écart-type	1,783	1,689	0,121	0,562	8,588	7,562

* = 16 mois

** = 10 mois

*** = 8 mois d'enquêtes.

Pour la période mai 57-février 58, avec des captures faites simultanément dans les zones témoin et DDT, la densité agressive a été réduite de 94% à l'intérieur des maisons et de 67% à l'extérieur. Le taux de piqûres a été six fois plus important à l'extérieur qu'à l'intérieur des maisons des villages traités au DDT alors qu'il était comparable dans la zone témoin.

**Figure 1:**
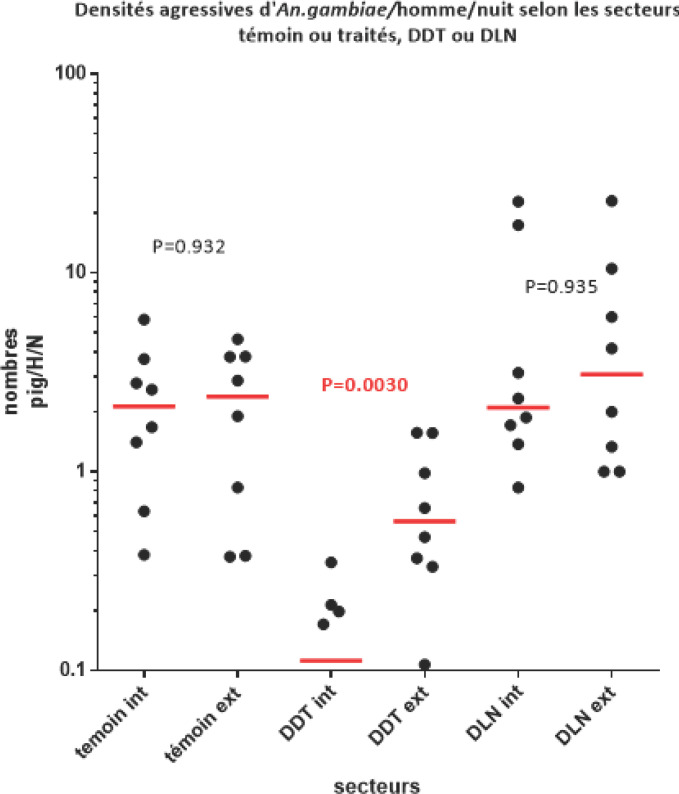
Nombre de piqûres (et médianes) d'*An. gambiae* enregistrées en captures de nuit sur sujets humains dans les secteurs témoins et traités au DDT ou la DLN Average number, and standard deviation, of landing rates of An. gambiae in night catches inside (int) and outside (ext) human habitations in control, DDT treated and DLN treated zones

En considérant les résultats obtenus simultanément dans les trois secteurs au 2^e^ semestre (pour pouvoir comparer des situations météorologiques comparables), il apparaît (Fig. 1) que les taux de piqûres d'*An. gambiae* ont été:

- comparables à l'intérieur et à l'extérieur des maisons des villages témoins (p = 0,932);
- six fois, et significativement, plus élevés à l'extérieur qu'à l'intérieur des maisons des villages traités au DDT ce qui met de nouveau en évidence l'effet irritant du produit;
- comparables à l'intérieur et à l'extérieur des maisons des villages traités à la DLN (p = 0,935);
- significativement plus élevés dans les maisons des villages traités à la dieldrine que dans ceux traités au DDT que ce soit à l'intérieur (p = 0,0002) ou à l'extérieur (p = 0,0042), traduisant bien l'impact de la résistance à ce produit. On note d'ailleurs des taux de piqûres en zone DLN égaux ou supérieurs à ceux de la zone témoin !

Ainsi, dans les zones traitées au DDT:

- le taux de piqûres a été réduit de 94% à l'intérieur et de 67% à l'extérieur par rapport à celui enregistré dans les zones témoins;
- le coefficient d'exophagie a été de 5,7 (alors qu'il est de 0,98 dans la zone témoin et de 0,95 dans la zone traitée à la dieldrine).

#### Infectivité (s)

Les résultats des dissections et examens des glandes salivaires pour la période de novembre 1956 à février 1958 (zone témoin versus zone DDT) et des 10 mois concomitants (mars 1957- février 1958) sont regroupés dans le tableau 3.

**Tableau III:** Indices sporozoïtiques d'*An. gambiae* dans les différentes parties de la zone pilote (toutes méthodes de captures regroupées) Sporozoitic index of An. gambiae in control, DDT treated and DLN treated zonesBobo-Dioulasso

Périodes	Indices	Témoin	DDT	DLN
nov. 56 - fév. 58	nb GS disséquées	2980	1269	
	nb GS+	89	3	
	i.s.	2,99%	0,24%	
mai 57 - fév. 58	nb GS disséquées	2804	1081	1949
	nb GS+	85	1	23
	i.s.	3,03%	0,092%	1,18%

GS = glandes salivaires; i.s. = indice sporozoïtique

Pour la période mai 57-février 58, les indices sporozoïtiques ont été réduits de 97% dans les maisons des villages traités au DDT et de 61% dans les villages de la zone traitée à la dieldrine par rapport aux villages de la zone témoin.

Pour cet indice d'infectivité, il est possible de considérer l'ensemble des échantillons quelle que soit la méthode de capture, car la comparaison « des indices sporozoïtiques moyens d'*An. gambiae* dans les différents types de captures dans les villages témoins en 1957 » a montré des valeurs comparables: 3,55% (n= 592) dans les captures de nuit à l'intérieur des maisons, 2,95% (n= 577) dans les captures de nuit à l'extérieur des maisons; 3,05% (n= 115) dans les captures de jour à l'intérieur des maisons; 2,74% (n= 475) dans les captures dans les cases-pièges et les moustiquaires-pièges ». Il faut retenir cette similitude des indices sporozoïtiques à l'intérieur et à l'extérieur des maisons, indiquant le maintien d'une transmission « résiduelle » à l'extérieur des habitations qui peut même être relativement accentuée par l'effet irritant du DDT augmentant l'exophilie d'*An. gambiae*.

#### Taux d'inoculation (h)

Si on considère les densités agressives (ma) et les indices sporozoïtiques (s) enregistrés pendant la période d'enquêtes simultanées dans les trois zones (mai-décembre 1957), il est possible d'avoir une certaine estimation, et de comparer, les taux quotidiens d'inoculation dans la zone témoin et les zones traitées pendant le deuxième semestre (tableau 4) avec la formule classique h= ma.s.

**Tableau IV:** Estimations des taux quotidien d'inoculation d'An. gambiae pour la période mai – décembre 1957 Estimation of An. gambiae *inoculation rate during May-December 1957*

	Témoin	DDT	DLN
ma_int_ ma_ext_	2,37 2,32	0,13 0,76	6,43 6,12
s	3,04% (n = 2761)	0,108% (n = 928)	0,905% (n = 1879)
h_int_ h_ext_	0,0721 0,0706	0,000143 0,00082	0,0582 0,0554

ma_int_= taux de piqûres à l'intérieur

ma_ext_= taux de piqûres à l'extérieur

h_int_ = taux d'inoculation à l'intérieur

h_ex_ = taux d'inoculation à l'extérieur

En considérant les valeurs dans leur globalité et leurs limites, pour la même période de l'année, il est possible d'estimer que dans les villages traités au DDT:

- le taux d'inoculation a été réduit de 99,8% dans les maisons et de 98,8% à l'extérieur par rapport aux villages témoins alors que la réduction n'est que, d'environ, 20% dans les villages traités à la dieldrine;
- le taux d'inoculation a été quasiment six fois plus important à l'extérieur qu'à l'intérieur (alors que les valeurs sont comparables en zones témoin et DLN).

Pour la période de huit mois considérée, les taux d'inoculation ont été de 17 piq. inf./Homme en zone témoin; 20-21 en zone DLN, mais de 0,03 à l'intérieur et à 0,2 à l'extérieur des maisons des villages traités au DDT, ce qui signifie une transmission particulièrement faible.

À partir de ces valeurs, on peut faire une estimation de la transmission de l'ordre de 25 piqûres infectées/Homme en zone témoin et 21 en zone DLN, démontrant la quasi-inefficacité de ce produit contre ce vecteur.

### Évolution d'*An. funestus*

#### Densités agressives (ma)

Les nombres moyens de piqûres d'*An. funestus* par Homme et par nuit dans les secteurs témoins et traités de la zone pilote sont regroupés dans le tableau 5 en tenant compte des différentes périodes de captures simultanées.

**Tableau V:** Nombres moyens de piqûres (ma) d'*An. funestus/*Homme/nuit dans les villages des trois secteurs, témoins, traités au DDT ou à la dieldrine Average number (and standard deviation) of landing rate of An. funestus/human being/night in control, DDT treated or dieldrin treated areas

Secteurs		Témoin		DDT		DLN	
Lieu		int	ext	int	ext	int	ext
de nov 56 à fév 58*	ma	2,903	3,366				
	écart type	3,344	5,448				
mai 57 à fév. 58**	ma	3,815	4,852	0,055	0,454		
	écart type	3,927	6,498	0,042	0,525		
mai 57 à déc. 57***	ma	4,300	5,841	0,062	0,539	0,083	0,201
	écart type	4,270	6,976	0,044	0,959	0,117	0,193

* = 16 mois

** = 10 mois

*** = 8 mois d'enquêtes.

**Figure 2:**
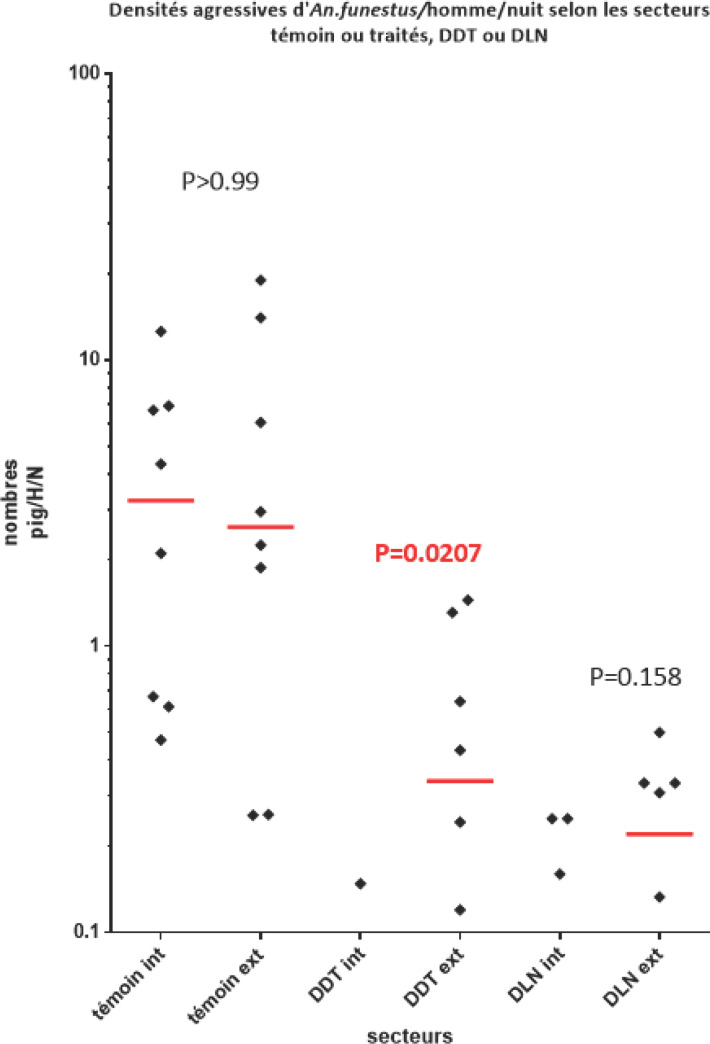
Nombre de piqûres (et médianes) d'*An. funestus* enregistrées en captures de nuit sur sujets humains dans les secteurs témoins et traités au DDT ou la dieldrine Average landing rate number (and median) of An. funestus in control, DDT treated and dieldrin treated areas

En considérant les résultats obtenus au 2^e^ semestre, alors que les captures se déroulent simultanément dans les différentes zones, il apparaît (Fig. 2) que les taux de piqûres d'*An. funestus* ont été:

- comparables à l'intérieur et à l'extérieur dans les villages de la zone témoin (p > 0,99);
- significativement plus élevés à l'extérieur qu'à l'intérieur dans les villages de la zone DDT (p = 0,021) où le taux de piqûres a été réduit de 98,5% dans les maisons et de 90,9% à l'extérieur par rapport à la zone témoin traduisant de nouveau l'efficacité et l'effet irritant du DDT;
- comparables à l'extérieur et à l'intérieur dans les villages traités à la dieldrine (p = 0,158) avec une réduction de 98,1% à l'intérieur et 96,5% à l'extérieur par rapport à la zone témoin, démontrant l'efficacité du produit sur ce vecteur;
- comparables dans les zones traitées au DDT et à la dieldrine aussi bien à l'intérieur (p = 0,585) qu'à l'extérieur (p = 0,221) démontrant la sensibilité conservée de cette espèce à ces deux produits.

Pour cette période, les coefficients d'exophagie (ext/int.) ont été de 1,35 en zone témoin, 8,7 en zone DDT confirmant de nouveau l'effet irritant du produit augmentant l'exophagie naturelle de cette espèce, et 2,42 en zone DLN qui a donc légèrement augmenté l'exophilie, connue, d'*An. funestus*.

#### Infectivité (s)

Pour l'année 1957, toutes méthodes de captures confondues, l'indice sporozoïtique moyen d'*An. funestus* a été estimé à 1,44% après dissections et observations de 4871 glandes salivaires avec des valeurs comparables quel que soit le mode de capture: 1,2% (n = 1083) en captures de nuit sur sujets humains dans les maisons et 2,13% (n = 1223) à l'extérieur; 1,22% (n = 2206) en captures de jour à l'intérieur et 1,11% (n = 359) en cases pièges et moustiquaires pièges.

Les résultats des dissections, toutes méthodes de captures confondues, pour la période novembre 1956-février 1958 concernant simultanément les secteurs témoins et DDT et pour la période juin-décembre 1957 concernant les trois secteurs simultanément, sont colligés tableau 6.

**Tableau VI:** Indices sporozoïtiques mensuels d'*An. funestus* dans les différentes parties de la zone pilote toutes méthodes de captures confondues Monthly sporozoitic index for An. funestus in the three sectors: control, DDT treated, dieldrin treated

Périodes	Indices	Témoin	DDT	DLN
nov. 56 - fév. 58	nb GS disséq.	6255	4842	30
	nb GS+	118	3	0
	i.s.	1,89%	0,062%	
juin - déc. 57	nb GS disséq.	4076	1372	29
	nb GS+	65	1	0
	i.s.	1,59%	0,073%	

GS = glandes salivaires; i.s. = indice sporozoïtique

Dans les zones traitées au DDT, l'indice sporozoïtique d'*An. funestus* a été réduit de 96,7% par rapport à la zone témoin pour l'ensemble de la période considérée et de 95% pour le deuxième semestre 1957.

#### Taux d'inoculation (h)

Si on isole les données concernant la période juin-décembre 1957, alors que les captures et les dissections ont effectivement été faites simultanément dans les trois secteurs, témoin, DDT et DLN (tableau 7), on peut estimer que:

- le taux d'inoculation a été réduit de 99,9% dans les maisons traitées au DDT et de 99,6% à l'extérieur:
- la transmission pourrait être de l'ordre d'une vingtaine de piqûres infectées pour la période (essentiellement saison des pluies) considérée et de l'ordre de 30 à 40 piqûres infectées/Homme par an dans les zones témoins avec une transmission plus accentuée à l'extérieur; elle n'est quasiment plus observée (avec les moyens techniques de l'époque) dans les zones traitées.

**Tableau VII:** *An. funestus*: taux quotidien de piqûres sur sujets humains, infectivité (s) et taux d'inoculation (h) An. funestus: *daily landing rate on human beings, infectivity (s) and inoculation rate (h)*

	Témoin		DDT		DLN	
	Int	Ext	Int	Ext	Int	Ext
ma/H/N	4,85 (±4,3)	6,64 (±7,3)	0,071 (± 0,04)	0,604 (± 0,57)	0,095 (±0,12)	0,23 (±0,19)
s	65GS+/4076= 1,59%		1GS+/1372= 0,073%		0/29	
h/mois	2,32	3,18	0,0016	0,013		
h/période (7 mois)	16,2	22,2	0,011	0,093		
h/an (proxy)	27,8	38,1	0,019	0,16		

ma/H/N = ma/Homme/nuit

infectivité s = nombre de glandes salivaires trouvées positives/nombre de glandes salivaires disséquées-examinées et taux d'inoculations

h/période = nombre de piqûres infectées par mois (h/mois) pour la période considérée (h/7 mois) (juin-décembre) et proxy pour une année (h/an) (avec la dieldrine les effectifs sont trop faibles)

### Synthèse des résultats entomologiques

La synthèse des résultats entomologiques montre:

- la remarquable efficacité du DDT qui a réduit de 99,8% (et plus) le taux d'inoculation des deux vecteurs majeurs et de plus de 90% le taux de piqûres dans les maisons traitées par rapport aux maisons des villages témoins;
- l'effet irritant du DDT sur *An. gambiae* et sur *An. funestus* avec une augmentation importante des coefficients d'exophagie;
- l'inefficacité de la dieldrine sur *An. gambiae* (bien qu'une réduction de 20% de la transmission ait pu être estimée), par contre le produit a été parfaitement efficace contre *An. funestus*.

### Résultats parasitologiques

#### Enquêtes transversales

Vingt-trois enquêtes ont été réalisées entre mars 1953 et octobre 1957 avec la confection et la lecture microscopique de 14168 gouttes épaisses (GE), dont 6943 ont été trouvées avec des Plasmodium (soit un indice général d'endémicité de 49%) et 497 avec des gamétocytes, soit un indice gamétocytique général de 3,51% (tableau 8a).

**Tableau VIIIa:** Indices parasitologiques enregistrés dans la zone pilote pendant toute la durée du programme Parasitological index noticed in the pilot zone during the whole programme

Indicateurs	Dates	nb enquêtes	nb GE faites	nb P+	IE (%)	nb Y+	IY (%)
témoin	avril 56 - oct. 57	4	946	573	60,6%	31	3,28%
DDT annuel	mars 53 - oct. 57	7	11781	5648	47,9%	403	3,42%
DDT sélectif	juin 55 - oct. 57	6	526	253	48,1%	18	3,43%
dieldrine	juin 55 -oct. 57	6	915	469	51,3%	45	4,93%
total		23	14168	6943	49,0%	497	3,51%

nb GE = nombre d'examens; nb P+ = nombre d'examens révélant des Plasmodium; IE = indice d'endémicité; nb Y+ = nombre d'examens révélant des gamétocytes; IY = indice gamétocytique)

Quatre enquêtes seulement ont été faites aux mêmes dates (avril et octobre 1956 et 1957) dans les quatre zones avec la confection de 8188 gouttes épaisses dont 3327 ont été trouvées positives (IE= 40,6%) et 251 avec des gamétocytes (IY = 3,07%) (tableau 8b).

**Tableau VIIIb:** Enquêtes parasitologiques faites aux mêmes périodes dans les différentes zones, témoins ou traitées Parasitological surveys done simultaneously during the same time in the control, DDT or dieldrin treated areas

	nb GE	nb P+	I.E. (%)	nb Y+	IY (%)
témoin	946	573	60,6%	31	3,28%
DDT annuel	6363	2397	37,7%	190	2,99%
DDT sélectif	342	129	37,7%	7	2,05%
dieldrine	537	228	42,5%	23	4,28%
total	8188	3327	40,6%	251	3,07%

I.E. = indice d'endémicité

L'examen de ce tableau montre que l'indice d'endémicité (IE):

- a été significativement plus faible dans les zones traitées (IE = 38,0% n= 7242) que dans la zone témoin (IE = 60,6% n = 946) (X^2^= 176,3 OR= 0,39 [0,35-0,46] différence -37%);
- a été comparable dans les zones traitées DDT «annuel» ou «sélectif», respectivement IE = 37,7% n = 6363 et IE = 37,7% n = 342 (X^2^ = 0,00035 p = 0,99 OR = 0,99 [0,79-1,25]);
- a été significativement plus faible dans la zone traitée au DDT (IE = 37,7%; n= 6705) que dans la zone traitée à la dieldrine (IE = 42,5% n= 53); (X^2^ = 4,83 p= 0,028 OR= 0,82 [0,68-0,98]).

Ainsi les traitements des maisons ont eu un impact significatif dans la réduction de la prévalence plasmodiale chez les enfants âgés de 2 à 9 ans et le DDT a été significativement meilleur que la DLN.

Par ailleurs, il faut remarquer que les indices gamétocytiques:

- ont été comparables dans les zones témoins et traitées, respectivement 3,28% (n = 946) et 3,04% (n = 7242) (X^2^ = 0,16 p = 0,69 OR = 1,08 [0,74-1,58]);
- ont été comparables dans les zones traitées au DDT « annuel » ou « sélectif », respectivement 2,99% (n = 6363) et 2,05% (n = 342) (X^2^ = 1,00 p = 0,32 OR = 1,47 [0,69-3,16]);
- ont été comparables dans les zones traitées au DDT ou à la DLN, respectivement 2,94% (n = 6705) et 4,28% (n = 537) (X^2^ = 3,05 p = 0,080 OR = 0,68 [0,44-1,05]).

La lutte antivectorielle par aspersions pariétales intradomiciliaires de DDT ou DLN ne paraît donc pas avoir réduit le « potentiel infectant » des enfants vis-à-vis des vecteurs et cette information participe à l'explication du maintien de la transmission, et la non-atteinte d'un des deux objectifs de l'éradication: l'assèchement du réservoir de parasites.

En outre, il a été effectué 15950 palpations de rates simultanément à la confection des gouttes épaisses pendant les 23 enquêtes du programme et 7431 rates ont été perçues, soit un indice splénique moyen de 46,6%, variable selon les zones (tableau 9a) et qui peut être rapproché de l'indice d'endémicité général (IE= 49,0%).

**Tableau IXa:** Indices spléniques chez les enfants de 2–9 ans enregistrés pendant toute la durée du programme (R+ = rate palpable sans tenir compte ici de la taille R1, R2) Splenic index in 2–9 years old children during the whole programme (R+=palpable spleen without considering the size)

Indicateur/secteurs	nb examens	R+	Indice splénique
témoin	979	825	84,3%
DDT annuel	13394	5648	42,2%
DDT sélectif	614	314	51,1%
Dieldrine	963	644	66,9%
total	15950	7431	46,6%

En ne considérant que les résultats des quatre enquêtes menées aux mêmes périodes (avril et octobre 1956 et 1957), l'indice splénique général a été de 48,4% (n = 9899) lui aussi variable selon les zones (tableau 9b).

**Tableau IXb:** Indices spléniques chez les enfants de 2–9 ans enregistrés pendant les quatre enquêtes faites simultanément dans les différentes zones Splenic index of 2–9 years old children during the 4 surveys silultaneously done in the differents sectors

Indicateur/secteurs	nb examens	R+	Indice splénique
témoin	979	825	84,3%
DDT annuel	7877	3411	43,3%
DDT sélectif	430	186	43,3%
Dieldrine	613	367	59,9%
total	9899	4789	48,4%

Il apparaît alors que les indices spléniques ont été:

- significativement plus faibles dans les zones traitées (44,4% n = 8920) que dans les zones témoins (84,3% n = 979) (X^2^ = 560,4 OR = 0,15 [0,12-0,18], différence = –47%);
- parfaitement comparables dans les zones traitées au DDT annuel (43,3% n= 7877) et DDT sélectif (43,3% n = 430) (X^2^ = 0,0004 p = 0,98 OR = 1,00 [0,82-1,22]);
- significativement plus faibles dans les zones traitées au DDT que dans les zones traitées à la DLN, respectivement 43,3% (n = 8307) et 59,9% (n = 613) (X^2^ = 63,5; OR = 0,51 [0,43-0,60], différence = -28%).

**Figure 3:**
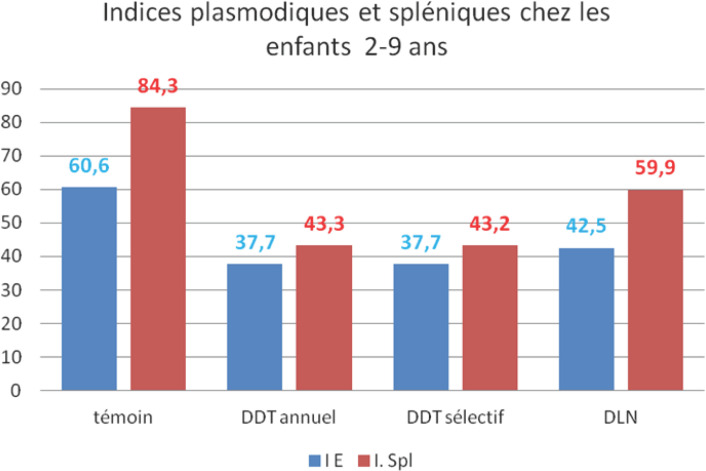
Indices d'endémicité (I.E.) et indices spléniques (I.Spl.) enregistrés simultanément dans les différents secteurs de la zone pilote Endemicity Index and splenic index noticed simultaneously in the various sectors of the pilot zone

Il est intéressant de souligner que les informations concernant l'évolution des indices spléniques recoupent celle des indices d'endémicité avec des valeurs significativement plus élevées dans les zones témoins que dans les zones traitées et dans les zones traitées à la DLN par rapport à celles traitées au DDT, que celui-ci ait été appliqué en mode annuel ou semestriel, général ou « sélectif » (Fig. 3).

### Indices de contaminations nouvelles (ICN) des enfants

Entre juin 1956 et février 1958, les Plasmodium ont été recherchés:

- 504 examens de nourrissons de 0 à 3 mois dans la zone témoin et 2445 dans la zone DDT;
- 231 examens de nourrissons 4 à 6 mois dans la zone témoin et 2279 dans la zone DDT;
- 100 examens de nourrissons de 7 à 12 mois dans la zone témoin et 3994 dans la zone traitée au DDT.

**Figure 4:**
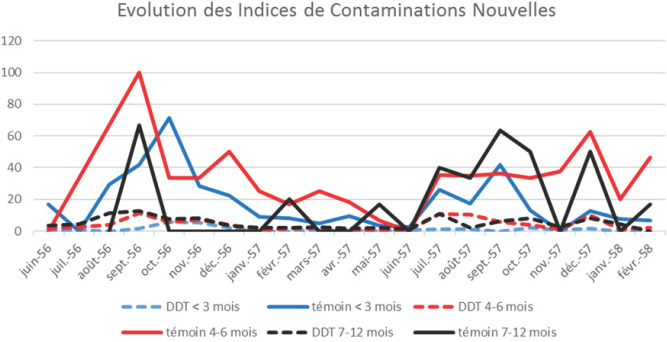
Variations mensuelles des indices de contaminations nouvelles chez les nourrissons de 0-3 mois, 4-6 mois et 7-12 mois des zones témoin et traitées au DDT Monthly variation of new contamination index in infants 0-3 months; 4-6 months; 7-12 months in control and DDT treated areas

Les examens des indices de contaminations nouvelles enregistrées simultanément dans la zone témoin et traitée, chaque mois, entre juin 1956 et février 1958, mettent clairement en évidence l'effet des traitements pariétaux intradomiciliaires au DDT sur la réduction de l'incidence de l'infestation plasmodiale chez les enfants de moins d'un an et indiquent bien les poussées saisonnières de transmission avec les pluies et la diminution régulière en saison sèche dans les zones non traitées (Fig. 4).

**Figure 5:**
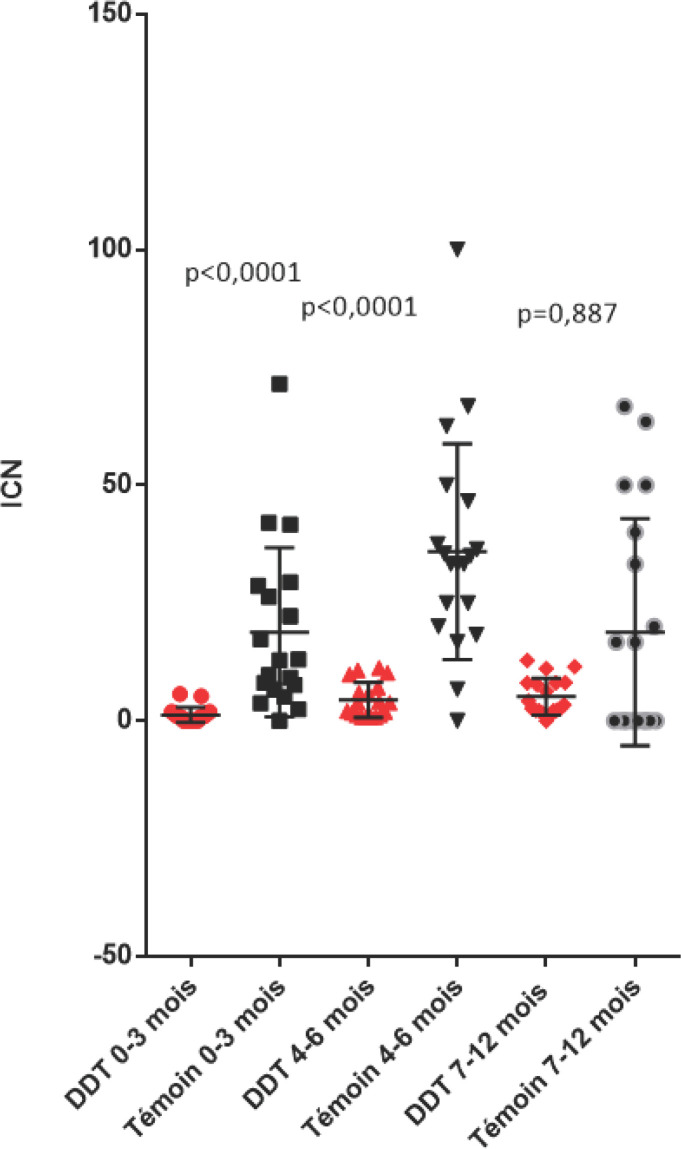
Indices de contaminations nouvelles chez les nourrissons des trois classes d'âge dans les zones traitées au DDT et les zones témoins (moyenne et écart-type) New infections index in infants of all three age groups in DDT-treated and control areas (mean and standard deviation)

Par ailleurs, la nouvelle analyse des données avec le logiciel Graph Pad et le test non paramétrique de Mann-Whitney (Fig. 5) révèle une réduction significative des ICN dans les zones DDT par rapport à la zone témoin chez les nourrissons 0-3 mois (p<0,0001) et 4-6 mois (p<0,0001), mais pas significative chez les nourrissons 7-12 mois (p= 0,887), traduisant une effective et importante réduction de la transmission mais pas son arrêt complet.

On peut aussi calculer que dans la zone traitée au DDT, les ICN sont significativement différents entre les nourrissons 0-3 mois et 4-6 mois (p = 0,0002), entre les 0-3 mois et 7-12 mois (p < 0,0001) mais pas entre les 4-6 mois et 7-12 mois (p = 0,2632).

### Indices cumulatifs

**Figure 6:**
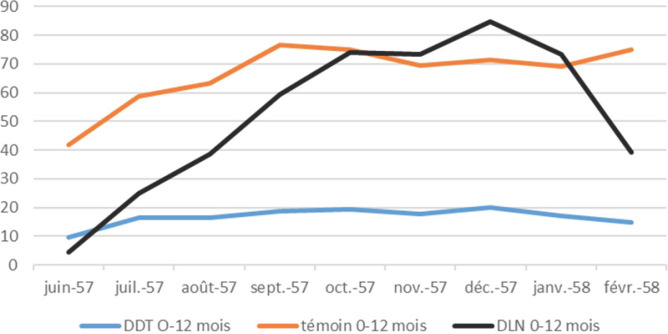
Évolutions mensuelles des indices cumulatifs chez les nourrissons de 0 à 12 mois dans les villages témoins et les villages traités au DDT ou la dieldrine Monthly evolution of cumulative index in 0-12 months infants of the control, DDT or DLN treated villages

Si on isole les données des indices cumulatifs calculés simultanément pour les trois zones (témoin, DDT, DLN) pendant la période juin 1957 - février 1958 (Fig. 6), il apparaît nettement qu'une certaine protection a été obtenue chez les enfants de moins d'un an avec le DDT (en termes quantitatifs, mais aussi l'absence de la poussée saisonnière avec les pluies), mais pas avec la DLN où l'effet cumulatif avec la poussée de transmission pendant les pluies est bien visible.

## Discussion

La zone pilote de lutte antipaludique de Bobo-Dioulasso a été créée en 1952 (avec le début des opérations le 1^er^ avril 1953) « afin de mettre au point les méthodes de lutte antipaludique les plus efficaces et les moins coûteuses en zone rurale de savanes soudaniennes » [18]. Elle était basée sur les aspersions pariétales intradomiciliaires de DDT (2,2 gr m.a./m^2^) ou de DLN (0,5 m.a./m^2^) ou de HCH (0,11 gr m.a./m^2^) avec une évaluation essentiellement entomologique, mais comprenant aussi conceptuellement, un volet médical et parasitologique pour confirmer l'arrêt espéré de la transmission.

Cette zone pilote s'inscrivait dans le concept de l'éradication dont un des buts était l'arrêt complet de la transmission. Le 6^e^ rapport du Comité d'experts sur le paludisme [46] avait donné la définition suivante: l'éradication du paludisme signifie la fin de la transmission et l'élimination du réservoir des cas infectants avec une campagne limitée dans le temps et réalisée avec un tel degré de perfection que lorsqu'elle se termine, il n'y a pas de reprise de la transmission. On peut souligner les deux objectifs majeurs et complémentaires (ce qui semble avoir été souvent oublié): arrêt de la transmission et élimination du réservoir de cas infectants, autrement dit les gamétocytes.

Pour Pampana [31], le terme éradication signifie ôter les racines (uprooting) et l'éradication du paludisme signifie l'extermination des parasites de la population humaine à grande échelle, mais pas celle des vecteurs. On voit là le concept d'anophélisme sans paludisme qui doit être considéré comme correct d'un point de vue écologique et de santé publique.

Avec la disponibilité, l'efficacité et la relative facilité d'emploi du DDT [28], l'accent a été essentiellement mis sur la lutte antivectorielle contre les stades adultes pour obtenir l'éradication alors que le volet élimination du réservoir se heurtait aux problèmes de la difficulté d'emploi des gamétocytocides ailleurs que dans un centre de santé et de détection des porteurs de gamétocytes qui sont asymptomatiques.

Le programme éradication se différencie alors du programme contrôle qui a pour objectif la réduction du paludisme à un niveau tel qu'il ne soit plus un problème majeur de santé publique. Bien que le 6e Comité d'experts n'ait pu définir ce qu'était « un problème majeur de santé publique », il a été admis [1] que la valeur de 10 cas de paludisme pour une population de 10000 personnes serait le seuil critique au-delà duquel le paludisme serait considéré comme un problème de santé publique.

Il est intéressant de souligner les considérations de l'époque. Après les succès des opérations d'aspersions intradomiciliaires (avec du DDT) notamment en Italie [11], il a été observé que « lorsque le paludisme n'est pas mortel, il disparaît du système circulatoire des patients généralement en moins de trois ans après le début de l'infection, même sans traitement ». On peut en conclure que si une grande zone est efficacement traitée au DDT pendant trois ans, il est possible d'arrêter les aspersions intradomiciliaires dès la 4ème année si un système de dépistage et traitement des cas est opérationnel. C'est le concept d'éradication [31], mais les traitements insecticides peuvent se poursuivre une 4ème voire une 5e année si nécessaire pour arriver à l'arrêt complet de la transmission.

C'est donc avec cet objectif de stopper la transmission en un laps de temps relativement réduit (3, 4, éventuellement 5 ans) qu'a été élaboré, réalisé et évalué le programme de la zone pilote de Bobo-Dioulasso. La biologie des vecteurs dans la région de Bobo-Dioulasso (mais pas dans la zone pilote) avait fait l'objet de nombreuses études [13, 14, 19] portant notamment sur l'exophagie, l'anthropophilie et les cycles d'agressivité des vecteurs majeurs et secondaires (dont le rôle ne doit pas être négligé dans les actions de lutte antivectorielle).

Les opérations de traitements des maisons de la zone pilote se sont échelonnées d'avril 1953 à février 1958 (on voit la durée de cinq ans d'un programme d'éradication) avec différents produits (tous organochlorés), selon différents protocoles (traitement total ou aspersion sélective sur le haut des murs et les plafonds) et concernant un nombre de villages variable selon les années et les possibilités, avec plusieurs méthodes d'échantillonnage des populations anophéliennes. En comparant les résultats de différentes méthodes de capture [18], il a été observé que *An. gambiae* et *An. funestus* manifestent pendant cette période (seconde moitié de la saison des pluies) une exophilie délibérée intense dès le stade femelle gorgée, cette exophilie étant plus nette chez *An. funestus* que chez *An. gambiae*. Nos observations sur *An. gambiae* rejoignent celles dans le Sud-Cameroun [26], où il a été observé une exophilie délibérée chez 18 à 50% des femelles gorgées selon les périodes. C'est là une importante observation avec une accentuation de cette exophilie induite par l'effet irritant du DDT.

Toutes les données des captures ont été fidèlement rapportées et analysées, à l'époque, avec une optique d'éradication. Avec l'autorisation personnelle du premier auteur (J. Hamon), elles ont été reprises avec une vision plus neutre et un certain recul en fonction de l'histoire observée de la lutte antivectorielle depuis cette période, notamment le quasi-arrêt de la lutte antivectorielle avec l'abandon du programme mondial d'éradication (1969) [11] jusqu'au Congrès d'Amsterdam (1992) identifiant la lutte antivectorielle comme une des méthodes de prévention.

Deux informations sont à retenir concernant les insecticides employés dans la zone pilote:

- la DLN fait partie des polluants organiques persistants (POP) de l'annexe A (chapitre Elimination) de la Convention de Stockholm [4] indiquant que sa production et son utilisation sont désormais prohibées. Le DDT est indiqué à l'annexe B (chapitre Restriction) avec pour « But acceptable pour la production et l'utilisation »: « Utilisation pour la lutte antivectorielle conformément à la deuxième partie de la présente annexe » (« La production et l'utilisation du DDT sont éliminées (…) Chaque Partie qui produit et/ou utilise du DDT limite cette production et/ou cette utilisation à la lutte contre les vecteurs pathogènes conformément aux recommandations et lignes directrices de l'Organisation mondiale de la santé relatives à l'utilisation du DDT et ce, pour autant que la Partie en question ne dispose pas de solutions de rechange locales sûres, efficaces et abordables »;
- le DDT est toujours utilisé en aspersions pariétales intradomiciliaires avec une grande efficacité, pour stopper l'épidémie de paludisme à Madagascar [5, 20, 39] ou éviter une épidémie au KwaZuluNatal et dans plusieurs pays d'Afrique australe [24].

L'équipe chargée du programme de lutte antivectorielle dans la zone pilote de Bobo-Dioulasso était composée de deux médecins et trois entomologistes médicaux et il est intéressant de noter cette association conceptuelle d'un volet entomologique et d'un volet parasitologique concernant des indicateurs classiques (indices spléniques, indices plasmodiques) et nouveaux (indice de contamination nouvelle, indice cumulatif) alors qu'à l'époque les études étaient souvent sectorisées.

Le programme a rencontré de nombreuses difficultés méthodologiques bien identifiables et identifiées. Par exemple, « pour les enquêtes paludométriques, l'échantillonnage des villages contrôlés n'a pas été pratiqué selon les méthodes statistiques, mais leur répartition et leur caractère hétérogène nous ont paru assez représentatifs de la diversité de la zone pilote » [18].

Par ailleurs, il est noté que « la numération des parasites était grossièrement évaluée de la façon suivante, à l'immersion 1/12 ou à 1/15: très rares (TR): quelques parasites après cinq minutes d'examen d'une goutte épaisse; rares (R): moins de un parasite pour cinq champs microscopiques; nombreux (N): quelques parasites par champ microscopique; très nombreux (TN): plus de cinq parasites par champ sans qu'il soit possible de savoir d'où vient cette méthode d'évaluation de la densité parasitaire. Il aurait été intéressant d'observer l'impact de la lutte antivectorielle sur la densité parasitaire, qui apparaît désormais comme un indicateur particulièrement pertinent en zone de paludisme stable, mais les résultats de cette étude ne sont pas disponibles.

Du fait de certaines contraintes opérationnelles, notamment l'accessibilité, certains villages ont été traités, ou non, d'une année à l'autre et « il n'existe pas à proprement parler de zone témoin puisque les villages témoins représentent des agglomérations situées en bout d'axes routiers, au-delà de la zone traitée » [18].

Néanmoins, il faut souligner la similitude de certains indicateurs entomologiques comme l'indice d'infectivité des vecteurs à l'époque et plusieurs années plus tard. Au cours de ce programme dans la zone pilote des villages des environs de Bobo-Dioulasso, les indices sporozoïtiques estimés par dissections et examens microscopiques des glandes salivaires, ont été de 3,55% (n = 592) pour *An. gambiae* et 1,20% (n = 1083) pour *An. funestus* en captures de nuit sur sujets humains dans les maisons en 1957.

Plus tard, en 1983-84, des indices de 4,8% (n = 659) et 4,6% (n = 303) ont été rapportés respectivement pour *An. gambiae* et *An. funestus* dans les villages de savane de Dandé-Tago, de 1,7% (n = 2072) et 2,1% (n = 1601) à Kongodjan avec des valeurs plus faibles en zones rizicoles de 0,5% (n = 8042), et 0,6% (n = 492) pour les deux vecteurs majeurs [37] ainsi que de 5,6% (n = 805) et 4,3% (n = 736) à Karankasso avant l'installation des moustiquaires imprégnées [36] et de 0,19% (n = 525) pour *An. gambiae* dans des quartiers du centre-ville de Bobo-Dioulasso [35].

Si on considère les valeurs rapportées de la zone pilote pour *An. gambiae* avec des taux d'inoculation de 0,058 piq.inf./H/nuit à l'intérieur et 0,048 piq.inf./H/nuit à l'extérieur en zone témoin, on peut alors estimer les taux mensuels d'inoculation respectivement à 1,74 piq.inf./H/ mois et 1,44 piq.inf./H/mois, soit un proxy de l'ordre de 21 piq.inf./H/an à l'intérieur et 17 à l'extérieur.

Pour *An. funestus*, les valeurs sont alors respectivement de 0,036 piq.inf./H/nuit soit 1,08 piq.inf./H/mois et # 13 piq.inf./H/an à l'intérieur d'une part et 0,049 piq.inf./H/nuit; 1,47 piq.inf./H/mois et # 18 piq.inf./H/an à l'extérieur.

Soit des valeurs annuelles de l'ordre de 34 à 35 piqûres infectées à l'intérieur ou l'extérieur des maisons dues à ces deux vecteurs majeurs. Cet ordre de grandeur des taux d'inoculation a été retrouvé bien plus tard, par exemple dans le village de Dandé-Tago avec des valeurs de l'ordre de 38 piq.inf./an dues à *An. gambiae* et 17 dues à *An. funestus*, mais avec d'importantes variations selon l'écologie locale puisqu'atteignant quelque 70 piq.inf./an dans le village de Kongodjan avec un marigot permanent et des totaux de l'ordre de 116 à 370 piq.inf./H/an à Karangasso (avec de grandes variations d'un quartier à l'autre voire d'une maison à l'autre). Cette grande hétérogénéité avait été remarquée, et soulignée, à l'époque de la zone pilote.

## Conclusion

Malgré les difficultés techniques rencontrées, les opérations d'aspersions intradomiciliaires ont pu être réalisées et les évaluations menées de façon rigoureuse dans la zone pilote de Bobo-Dioulasso. Il est alors apparu un impact significatif des actions de lutte sur la réduction de la densité anophélienne agressive pour l'Homme et les taux d'inoculation, mais aussi un certain maintien, bien que minime, de la transmission, traduite en termes entomologiques et parasitologiques. Il en a été conclu à un «semi-échec », induisant un changement radical de stratégie de lutte contre le paludisme, la lutte antivectorielle se consacrant alors à la recherche et l'évaluation de nouveaux insecticides, notamment avec la station expérimentale de Soumousso (J. Hamon com. pers.) devenu Centre Collaborateur de l'OMS pour l'évaluation des insecticides, et où ont été expérimentées les premières moustiquaires imprégnées de perméthrine 30 ans plus tard.

Une autre analyse des résultats obtenus dans la zone pilote de Bobo-Dioulasso peut permettre une conclusion plus positive si on admet une conception de la lutte visant à une réduction et l'élimination progressive du paludisme plutôt qu'une éradication « radicale » avec une seule méthode de lutte et une opération limitée dans le temps selon le modèle classique.

En effet, si on se centre sur les résultats comparables en termes de méthodes d'évaluation et de périodes d'enquêtes simultanées dans les trois zones: témoin, traitée au DDT et traitée à la DLN, il apparaît une remarquable efficacité des traitements (surtout avec le DDT) qui ont permis de réduire de plus de 90% les taux de piqûres des vecteurs majeurs et de 99% les taux d'inoculation. Tout programme qui obtiendrait actuellement un tel résultat serait considéré comme une grande réussite. Par exemple, lors de la première évaluation des moustiquaires imprégnées de deltaméthrine à l'échelle du village, en zone de savane, à Karankasso, il a été obtenu une réduction de 88% du taux d'inoculation dû à *An. gambiae* et 92% pour *An. funestus*.

Plusieurs enseignements peuvent être tirés de ces opérations de lutte antivectorielle dans la zone pilote de Bobo-Dioulasso et gardés en mémoire.

Ce programme a abordé trois des questions majeures soulevées à l'époque, et apporté des réponses positives à chacune:

- faisabilité: est-il possible de réaliser des opérations d'aspersions intradomiciliaires à grande échelle et pendant une certaine durée (plusieurs années) en zone rurale d'endémie palustre en Afrique sub-saharienne? Le projet a effectivement duré cinq ans, couvert plusieurs milliers de kilomètres carrés et protégé plusieurs milliers de personnes. Un programme de ce type était donc faisable.
- efficacité entomologique:
- est-ce que la lutte antivectorielle pouvait réduire les nombres de piqûres et l'intensité de la transmission même si le vecteur majeur présentait un certain degré de résistance à l'un des produits? La réponse a été positive avec une réduction de l'infectivité d'*An. gambiae* avec la DLN, malgré la résistance (mais le taux d'inoculation n'a pas été que faiblement impacté);
- est-ce que le DDT était une arme de grande efficacité pour réduire la transmission due aux deux vecteurs majeurs qu'étaient *An. gambiae* et *An. funestus*? La réponse a été encore positive avec une diminution de plus de 99% du taux d'inoculation.
- est-ce que le DDT avait un effet particulièrement irritant sur les vecteurs? La réponse était positive avec une augmentation du taux de piqûres à l'extérieur des habitations traitées.
- efficacité parasitologique: est-ce que la lutte antivectorielle basée sur les aspersions intradomiciliaires de DDT (et DLN) permettait de réduire l'impaludation des enfants? La réponse peut alors être plus nuancée. Il a quand même été noté une réduction d'un tiers de l'indice plasmodique des enfants (2–9 ans) et de la moitié des indices spléniques dans ce groupe d'âge.

Le fait que 40% des enfants soient encore porteurs, asymptomatiques, de Plasmodium et 40% avec des rates palpables ne permet cependant pas de considérer le programme comme un succès complet à ce niveau mais encourageant tout de même.

Par ailleurs, le taux d'incidence a été significativement réduit chez les nourrissons (0-6 mois), mais « ous les nouveau-nés étaient infectés dans l'année» (J. Hamon com. pers.) traduisant là aussi le maintien de la transmission, même à un bas niveau.

En outre, les indices gamétocytiques sont restés du même ordre de grandeur signant la persistance d'un réservoir de parasites pour le maintien de la transmission ce qui pose le problème en termes de possibilités « d'assèchement du réservoir de virus » tel que recommandé dans le programme d'éradication du paludisme.

Dans ces conditions et malgré ces réponses positives, le programme avait été considéré à l'époque comme un «semi-échec» car l'objectif transmission zéro n'était pas atteint et que «le paludisme continue à être transmis » dans cette zone comme dans d'autres en Afrique tropicale. Pour Hamon et al, «dans l'ensemble, les résultats (des zone pilotes) sont désappointants. Presque toutes les zones pilotes et campagnes sont interrompues à l'heure actuelle».

On peut, et on doit s'interroger sur les raisons de ces résultats, et leurs interprétations. Pour Vaucel (Inspecteur général des Instituts Pasteurs d'Outre-Mer), dans la préface de l'article relatant les résultats de la zone pilote de Bobo-Dioulasso, « les raisons du semi-échec sont clairement mises en évidence: exophagie de *An. gambiae* encore accentuée par l'effet répulsif de l'insecticide, exophagie de An.nili que son exophilie met à l'abri de l'effet toxique, exophagie encore, mais à un degré moindre, de *An. funestus* et An.coustani, rôles des espèces secondaires, gène de résistance à la dieldrine chez *An. gambiae* ». Du fait de cette résistance, ainsi qu'au HCH, ces produits ont été abandonnés pour les traitements des maisons. Les causes entomologiques de ces résultats considérés « dans l'ensemble médiocres » ont été bien identifiées et elles peuvent être très différentes selon les vecteurs, les biotopes etc. de sorte que les programmes de lutte doivent être adaptés aux conditions entomologiques, écologiques mais aussi socio-économiques, etc., de chaque situation nécessitant alors des études préalables précises et pluridisciplinaires.

On doit aussi considérer les comportements des populations humaines concernées par ces activités: acceptabilité d'équipes venant de l'extérieur et les problèmes, bien connus, soulevés par les aspersions intradomiciliaires entraînant souvent un refus des aspersions suivantes et les portes fermées; s'ajoutent les activités sociales le soir à l'extérieur des maisons donc des possibilités d'infections, les cases de culture habitées, de façon plus ou moins temporaire, pendant la saison agricole intense et les difficultés opérationnelles de leur traitement, etc. Des problèmes relationnels avaient été rencontrés au cours du programme de lutte antivectorielle dans la zone pilote et des solutions avaient été apportées (avec des « mesures de protection maternelle et infantile ») pour « regagner la confiance des populations » et poursuivre les opérations prévues. Les problèmes d'acceptabilité et de participation communautaire effective sont toujours d'actualité avec, par exemple, le maintien et l'entretien des moustiquaires imprégnées désormais distribuées à très large échelle, avec un succès certain.

En effet selon le rapport officiel du paludisme dans le monde en 2015 (29) pour la période 2001-2015 « en Afrique subsaharienne, les interventions antipaludiques expliquent 70% des 943 millions de cas de paludisme en moins entre 2001 et 2015, soit un total de 663 millions de cas évités (plage comprise entre 542 et 753 millions) dont 69% l'ont été grâce à l'utilisation de moustiquaires imprégnées d'insecticide (MII) (incertitude: 63%-73%), 21% grâce aux combinaisons thérapeutiques à base d'artémisinine (ACT) (incertitude: 17%;-29%) et 10% grâce aux pulvérisations intradomiciliaires d'insecticides à effet rémanent (PID) (incertitude: 6%-14%) ».

L'emploi généralisé des moustiquaires imprégnées aurait permis d'éviter quelques 457 millions de cas et les aspersions intradomiciliaires quelques 66 millions de cas, autrement dit la lutte antivectorielle aurait permis d'éviter environ 524 millions de cas entre 2001 et 2015 [29].

Pour une élimination progressive du paludisme et dans l'attente d'un vaccin opérationnel, la lutte antivectorielle conserve donc une place majeure en complément de l'accès au centre de santé, à un diagnostic rapide et fiable et à un traitement simple, adapté et efficace. Dès 1963, Hamon et al [17] préconisaient comme « remèdes » aux causes d'échec, de compléter l'action intense quoique insuffisante de la lutte antivectorielle par une chimiothérapie collective (selon différentes modalités et différents antipaludiques notamment la chloroquine seule ou associée à la primaquine) pour aboutir ainsi à l'arrêt complet de la transmission.

Enfin, ce projet pilote démontre, s'il en était besoin, que la lutte antivectorielle peut toujours être réalisée, malgré toutes les difficultés opérationnelles et techniques rencontrées, et que les résultats obtenus doivent être analysés de façon pragmatique pour le bénéfice des populations exposées au paludisme, et autres maladies parasitaires à transmission vectorielle.

## Remerciements

Nous tenons à remercier vivement Mr J. Hamon qui nous a permis de reprendre ses résultats et nous a fourni de nombreuses et judicieuses informations sur les travaux menés dans la zone pilote et les premières analyses des données obtenues.

Nous tenons à remercier aussi le Prof. J. Roux pour ses pertinentes observations et commentaires suscités par ce document avec son expérience de paludologue de terrain et de directeur du Centre Muraz de Bobo-Dioulasso à l'époque des premières moustiquaires imprégnées.

Nous remercions aussi vivement les lecteurs/lectrices pour leurs examens attentifs de notre document et leurs intéressants apports.

## Liens D'intérêt

Les auteurs déclarent de ne pas avoir de liens d'intérêt.

## Figures and Tables

**Figure:**
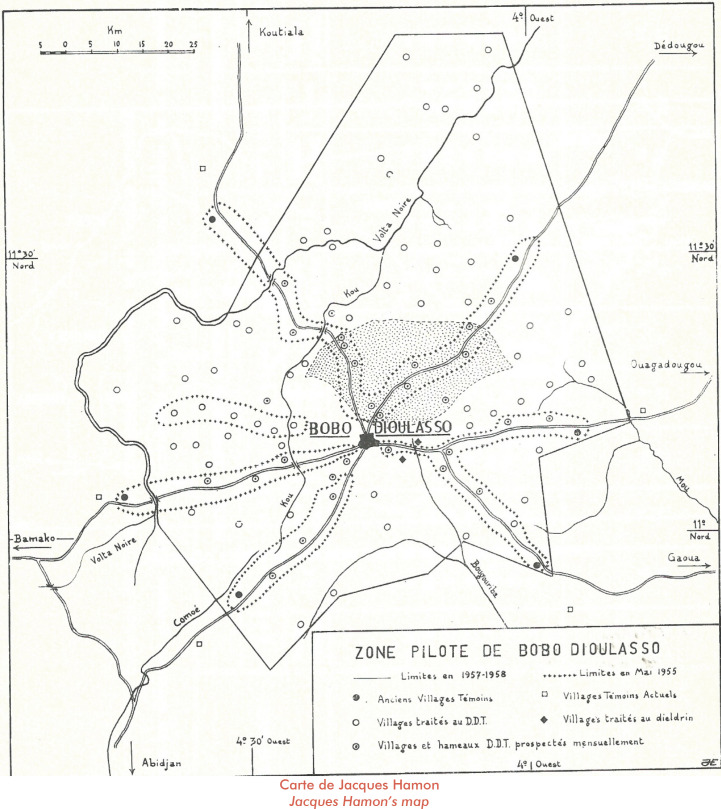
Carte de Jacques Hamon Jacques Hamon's map

## Annexe

### Historique des Aspersions dans la Zone Pilote (Extrait de Hamon J, Choumara R, Adam J, Bailly H, Ricossé J. Le Paludisme dans la zone pilote de Bobo Dioulasso Haute-Volta. Cahiers de L'orstom 1959, 1: 125 pp.)

#### Insecticides Utilisés

De 1953 à 1955 ont été employés le DDT et le HCH tous les deux sous forme de poudre mouillable à 75%.

À partir de 1955, l'HCH venant à manquer, les aspersions furent continuées au DDT seul et, pour deux villages jamais traités précédemment, au Dieldrin, sous forme de poudre mouillable à 50% et d'émulsion à 20%.

#### Dosages Utilisés

Le DDT a été employé à la dose de 2,2 grammes d'isomère p.p' au mètre carré, le HCH à la dose de 0,11 gramme d'isomère gamma par mètre carré, et le Dieldrin à la dose de 0,5 gramme de produit technique au mètre carré.

##### Modifications successives intervenues dans la zone pilote

Au début des opérations, le 1^er^ avril 1953, la zone s'étendait sur 2886 kilomètres carrés et était divisée comme suit en trois secteurs d'intérêts:

**Table:**

Secteur 1	Groupe 1 HCH trimestriel Groupe 2 HCH semestriel	8 villages 4 villages	4756 habitants 2088 habitants
Secteur 2	Groupe 1 DDT annuel Groupe 2 DDT semestriel	14 villages 12 villages	4491 habitants 6722 habitants
Secteur 3	DDT+HCH annuel	3 villages	765 habitants
	total	41 villages	18822 habitants

En 1954, six nouveaux villages furent inclus, si bien que la zone pilote comprenait alors 47 villages groupant 21633 habitants. Les mesures de désinsectisation concernaient les villages situés le long des axes routiers (cf. carte), les villages situés « en pleine brousse » n'étaient pas traités, pas plus que les cases de culture.

##### La zone pilote de Bobo-Dioulasso et son évolution (d'après Hamon et al., 1959)

En 1955, pour des raisons opérationnelles 15 villages éloignés (6570 habitants) n'ont plus été traités mais la désinsectisation a concerné 16 nouveaux villages et les cases de culture. « On comptait alors 48 villages groupant 21373 habitants pour une surface de 2069 kilomètres carré ». Deux de ces nouveaux villages ont été traités à la dieldrine et un troisième a été traité au DDT en « aspersion sélective annuelle » (traitement uniquement des plafonds et du haut des murs sur une bande de 80 centimètres). En 1955 il n'y a eu qu'une seule aspersion de DDT couvrant tous les villages et les cases de culture.

En 1956 une extension a été décidée («par la Mission conjointe O.M.S. Gouvernement Français »). Les traitements des villages situés sur les axes routiers et abandonnés et 1955 ont été traités ainsi que «tous les villages et cases de culture de brousse situés entre les tronçons routiers». « La nouvelle zone traitée a ainsi constitué une couronne de protection de largeur fort inégale tout autour de la zone rétrécie de mai 1955 ».

En février 1958 la zone pilote comportait, sur 7347 kilomètres carrés: « A l'origine, cinq villages témoins avaient été choisis à la périphérie de la zone traitée, mais peu à peu l'absentéisme devint important lors des contrôles mensuels et semestriels et ils furent traités en 1954. Cinq nouveaux villages témoins furent immédiatement choisis pour les enquêtes entomologiques. En 1955, des mesures de protection maternelle et infantile ayant permis de regagner la confiance de la population, les contrôles paludométriques furent repris dans des villages non traités ». « Actuellement six villages servent de témoins pour les enquêtes paludométriques, quatre qui n'ont jamais été traités et deux précédemment traités mais abandonnés en 1955; trois seulement de ces villages sont les mêmes que ceux servant au contrôle entomologique ».

**Table:**

Secteur 1 DDT annuel	85 villages	37537 habitants
Secteur 2 DD semestriel	18 villages	9074 habitants
Secteur 3 DDT sélectif annuel Dieldrin annuel	1 village 2 villages	2113 habitants 1185 habitants
Total	106 villages	49909 habitants

## References

[1] Bruce-Chwatt LJ, Malaria research and eradication in the USSR. (1959). A review of Soviet achievements in the field of malariology. Bull World Health Organ.

[2] Carnevale P, Mouchet J, La lutte antivectorielle au Cameroun. (2001). Passé-présent-avenir. Réflexions. Bull Soc Pathol Exot.

[3] Carnevale P, Robert V, Boudin C, Halna JM, Pazart L, Gazin P, Richard A, Mouchet J (1988). La lutte contre le paludisme par des moustiquaires imprégnées de pyréthrinoides au Burkina Faso. Bull Soc Pathol Exot Filiales.

[4] Convention de Stockholm sur les polluants organiques persistants 2001. (2010). Texte et Annexes, telle qu'amendée en 2009. Secrétariat de la Convention de Stockholm sur les polluants organisques persistants. OME-PNUE Genève.

[5] Curtis CF (2002). Restoration of malaria control in the Madagascar highlands by DDT spraying. Am J Trop Med Hyg.

[6] Darriet F, Robert V, Tho Vien N, Carnevale P (1984). Evaluation de l'efficacité sur les vecteurs de paludisme de la permethrine en imprégnation de moustiquaires intactes et trouées. WHO/VBC/84899 & WHO/MAL/841008.

[7] Detinova TS (1963). Méthodes à appliquer pour classer par groupes d'âge les diptères présentant une importance médicale. OMS.

[8] Dobson MJ, Malowany M, Snow RW (2000). Malaria control in East Africa: the Kampala Conference and the Pare-Taveta Scheme: a meeting of common and high ground. Parassitologia.

[9] Draper CC, Smith An, Malaria in the Pare area of Tanganyika. (1960). Part II. Effects of three years' spraying of huts with dieldrin. Trans R Soc Trop Med Hyg.

[10] Draper CC, Lelijveld JL, Matola YG, White GB, Malaria in the Pare area of Tanzania. (1972). IV. Malaria in the human population 11 years after the suspension of residual insecticide spraying, with special reference to the serological findings. Trans R Soc Trop Med Hyg.

[11] Gramiccia G, Beales PF, Wernsdorfer WH, McGregor I, The recent history of malaria control and eradication. In: Malaria. (1988). Principles and Practice of Malariology. Churchill Livingstone Edinburgh London Melbourne and New York.

[12] Haddow AJ (1942). The mosquito fauna and climate of native huts at Kisumu, Kenya. Bull Ent Res.

[13] Hamon J (1954). Contribution à l'étude des Culicidés de la région de Bobo-Dioulasso (Haute-Volta). Ann Parasit hum comp.

[14] Hamon J, Adam JP, Grjebine An (1956). Observations sur la répartition et le comportement des anophèles de l'Afrique-Equatoriale Française, du Comeroun et de l'Afrique Occidentale. Bull World Health Organ.

[15] Hamon J, Eyraud M, Sales S (1958). Observations préliminaires sur la présence du géne de résistance au dieldrin chez *Anopheles gambiae* Giles dans des zones non traitées au dieldrin de la région de Bobo-Dioulasso (Haute-Volta, A.O.F. Bull Soc Pathol Exot Filiales.

[16] Hamon J, Choumara R, Eyraud M, Konate TAn (1957). Apparition dans la zone pilote de lutte antipaludique de Bobo-Dioulasso (Haute-Volta, A.O.F.) d'une souche d'*Anopheles gambiae* Giles (Diptères, Culicidés) résistante au Dieldrin. Bull Soc Pathol Exot Filiales.

[17] Hamon J, Mouchet J, Chauvet G, Lumaret. (1963). Bilan de quatorze années de lutte contre le paludisme dans les pays francophones d'Afrique tropicale et à Madagascar. Considérations sur la persistance de la transmission et perspectives d'avenir. Bull Soc Pathol Exot Filiales.

[18] Hamon J, Choumara R, Adam J, Bailly H, Ricossé J (1959). Le Paludisme dans la zone pilote de Bobo Dioulasso Haute-Volta. Cahiers de l'ORSTOM 1.

[19] Holstein M, Biologie d'*Anopheles gambiae*. (1952). Recherches en Afrique Occidentale Française. Ser Monogr Org mond Santé.

[20] Jambou R, Ranaivo L, Raharimalala L, Randrianaivo J, Rakotomanana F, Modiano D, Pietra V, Boisier P, Rabarijaona L, Rabe T, Raveloson N, De Giorgi F (2001). Malaria in the highlands of Madagascar after five years of indoor house spraying of DDT. Trans R Soc Trop Med Hyg.

[21] Lengeler C., Cattani J., Savigny Don de, Net Gain. (1996). A New Method for Preventing Malaria Deaths. Int Dev Res Centre, WHO.

[22] Livadas G, Mouchet J, Gariou J, Chastang R (1958). Peut-on envisager l'éradication du paludisme dans la région forestière du Sud Cameroun?. Riv Malariol.

[23] Macdonald G (1956). Epidemiological basis of malaria control. Bull World Health Organ.

[24] Mabaso ML, Sharp B, Lengeler C (2004). Historical review of malarial control in southern African with emphasis on the use of indoor residual house-spraying. Trop Med Int Health.

[25] Molineaux L, Gramiccia G, The Garki Project. (1980). Research on the epidemiology and control of malaria in the Sudan savanna of West Africa.

[26] Mouchet J, Gariou J (1957). Exophilie et exophagie d'*Anopheles gambiae* Giles 1902, dans le Sud Cameroun. Bull Soc Pathol Exot Filiales.

[27] Muench H (1959). Catalytic models in epidemiology.

[28] Müller P (1946). Über Zusammenhänge zwischen Konstitution und insektizider Wirkung. Helvetica Chimica Acta.

[29] OMS. (2016). Rapport sur le paludisme dans le Monde.

[30] OMS. (2017). Cadre pour l'élimination du paludisme.

[31] Pampana EAn (1963). Textbook of Malaria Eradication.

[32] Pampana E, Russell P (1955). Malaria- a World problem. Wld Hlth Org Chronicle.

[33] Polovodova VP (1949). [Détermination de l'âge physiologique d'*Anopheles* femelle]. Med Parazit (Mosc.).

[34] Robert V, Carnevale P (1991). Influence of deltamethrin treatment of bed nets on malaria transmission in the Kou valley, Burkina Faso. Bull World Health Organ.

[35] Robert V, Gazin P, Ouedraogo V, Carnevale P, Le paludisme humain à Bobo-Dioulasso (Burkina Faso). (1986). 1. Etude entomologique de la transmission. Cah ORSTOM, sér Ent méd Parasitol.

[36] Robert V, Carnevale P, Ouedraogo V, Petrarca V, Coluzzi M (1988). La transmission du paludisme humain dans un village de savane du sud-ouest du Burkina Faso. Ann Soc Belg Med Trop.

[37] Robert V, Gazin P, Boudin C, Molez JF, Ouedraogo V, Carnevale P (1985). La transmission du paludisme en zone de savane arborée et en zone rizicole des environs de Bobo Dioulasso (Burkina Faso). Ann Soc Belg Med Trop.

[38] Roberts FH, O'Sullivan PJ (1948). Studies on the behaviour of adult Australasian anophelines. Bull Entomol Res.

[39] Romi R, Razaiarimanga MC, Raharimanga R, Rakotondraibe EM, Ranaivo LH, Pietra V, Raveloson A, Majori G (2002). Impact of the malaria control campaign (1993-1998) in the highlands of Madagascar: parasitological and entomological data. Am J Trop Med Hyg.

[40] Smith An, Malaria in the Taveta area of Kenya and Tanganyika. (1962). II. Entomological findings three years after the spraying period. East Afr Med J.

[41] Smith An, Malaria in the Taveta area of Kenya and Tanzania. (1966). IV. Entomological findings six years after the spraying period. East Afr Med J.

[42] Smith A, Draper CC (1959). Malaria in the Taveta area of Kenya and Tanganyika. I. Epidemiology. East Afr Med J.

[43] Smith A, Pringle G, Malaria in the Pare area of Kenya and Tanzania. (1967). Part V. Transmission eight years after the spraying period. E Afr Med J.

[44] Wilson B. (1960). Report on the Para-Taveta Malaria Scheme 1954-1959. East African High Commission.

[45] WHO. (1951). Report on malaria conference in Equatorial Africa.

[46] WHO. (1957). Expert Committee on Malaria. Sixth Report Techn Rep Ser.

